# Spin–orbit torque-assisted switching in magnetic insulator thin films with perpendicular magnetic anisotropy

**DOI:** 10.1038/ncomms12688

**Published:** 2016-09-01

**Authors:** Peng Li, Tao Liu, Houchen Chang, Alan Kalitsov, Wei Zhang, Gyorgy Csaba, Wei Li, Daniel Richardson, August DeMann, Gaurab Rimal, Himadri Dey, J. S. Jiang, Wolfgang Porod, Stuart B. Field, Jinke Tang, Mario C. Marconi, Axel Hoffmann, Oleg Mryasov, Mingzhong Wu

**Affiliations:** 1 Department of Physics, Colorado State University, Fort Collins, Colorado 80523, USA; 2 MINT Center, University of Alabama, Tuscaloosa, Alabama 35401, USA; 3 Materials Science Division, Argonne National Laboratory, Lemont, Illinois, 60439, USA; 4 Department of Electrical Engineering, University of Notre Dame, Notre Dame, Indiana 46556, USA; 5 Engineering Research Center for Extreme Ultraviolet Science and Technology and Department of Electrical and Computer Engineering, Colorado State University, Fort Collins, Colorado 80523, USA; 6 Department of Physics & Astronomy, University of Wyoming, Laramie, Wyoming 82071, USA

## Abstract

As an in-plane charge current flows in a heavy metal film with spin–orbit coupling, it produces a torque on and thereby switches the magnetization in a neighbouring ferromagnetic metal film. Such spin–orbit torque (SOT)-induced switching has been studied extensively in recent years and has shown higher efficiency than switching using conventional spin-transfer torque. Here we report the SOT-assisted switching in heavy metal/magnetic insulator systems. The experiments used a Pt/BaFe_12_O_19_ bilayer where the BaFe_12_O_19_ layer exhibits perpendicular magnetic anisotropy. As a charge current is passed through the Pt film, it produces a SOT that can control the up and down states of the remnant magnetization in the BaFe_12_O_19_ film when the film is magnetized by an in-plane magnetic field. It can reduce or increase the switching field of the BaFe_12_O_19_ film by as much as about 500 Oe when the film is switched with an out-of-plane field.

## Abstract (web summary)

By virtue of strong spin-orbit coupling, a current-carrying heavy metal may generate a torque on the magnetization of an interfaced ferromagnetic metal. Here, the authors demonstrate how this effect assists the magnetic reversal of ferromagnetic insulators with perpendicular magnetic anisotropy.

## Graphical abstract

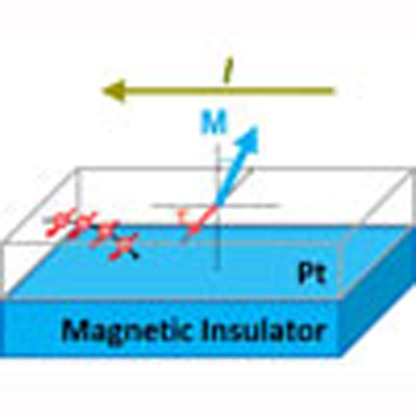

Magnetization switching in ferromagnetic materials is of both fundamental interest and technological significance. One way to switch the magnetization in a ferromagnetic film is to pass a spin-polarized electron current perpendicularly through the film. The spin-polarized current can be produced by passing a charge current through a spin polarizer, which is typically a hard magnetic layer separated from the target film by a normal metal spacer^1^,^2^,^3^,^4^. As the polarized electrons flow through the ferromagnetic film, they transfer spin angular momentum to the film and thereby produce a torque that can switch the magnetization in the film^5^,^6^. Magnetic memory based on such spin torques has already been commercialized in recent years.

The above-mentioned conventional spin-torque switching, however, has a limit, namely, that the angular momentum transferred per unit charge in the applied current usually cannot exceed a quanta of spin (*ħ*/2) (ref. 7). Fortunately, very recent work demonstrates that one can exceed this limit by the use of spin–orbit torque (SOT)^7^,^8^,^9^,^10^,^11^,^12^,^13^,^14^,^15^,^16^. The demonstration generally takes a non-magnetic heavy metal (HM)/ferromagnetic metal (FM) bi-layered structure, and makes use of spin–orbit coupling-produced spin Hall effect (SHE)^17^,^18^,^19^,^20^ in the HM film to convert an in-plane charge current to a pure spin current that flows across the HM thickness, produces spin accumulation at the HM/FM interface, and thereby exerts a torque on the FM. In this case, each electron in the applied current can undergo multiple spin-flip scattering at the interface, therefore enabling considerably more efficient switching than in the conventional spin-torque case.

In addition to breaking the limit of the conventional spin torque, the SOT mechanism also makes possible current-induced magnetization switching in low-damping magnetic insulators (MIs), which was impossible with the conventional spin-torque geometry. Although such a possibility has not been demonstrated so far, recent work has already demonstrated the use of SOTs from charge currents in a HM layer to either manipulate magnetic damping^21^,^22^ or induce magnetization precession^22^,^23^,^24^ in a neighbouring MI layer.

Here we report SOT-assisted switching in HM/MI systems. The experiments made use of Pt/BaFe_12_O_19_ bi-layered structures. Thanks to its strong spin–orbit coupling, Pt has been widely used to produce pure spin currents in previous studies^22^,^23^,^24^,^25^,^26^,^27^,^28^,^29^. BaFe_12_O_19_ is an M-type barium hexagonal ferrite and is often referred as BaM. It is one of the few MIs with strong magneto-crystalline anisotropy and shows an effective uniaxial anisotropy field of about 17 kOe^30^,^31^. It is found that the switching response in the BaM film strongly depends on the charge current applied to the Pt film. When a constant magnetic field is applied in the film plane, the charge current in the Pt film can switch the normal component of the magnetization (*M*_⊥_) in the BaM film between the up and down states. The current also dictates the up and down states of the remnant magnetization when the in-plane field is reduced to zero. When *M*_⊥_ is measured by sweeping an in-plane field, the response manifests itself as a hysteresis loop, which evolves in a completely opposite manner if the sign of the charge current is flipped. When the coercivity is measured by sweeping an out-of-plane field, its value can be reduced or increased by as much as about 500 Oe if an appropriate charge current is applied.

## Results

### Properties of Pt/BaM films and Hall bar structures

**Figure 1:**
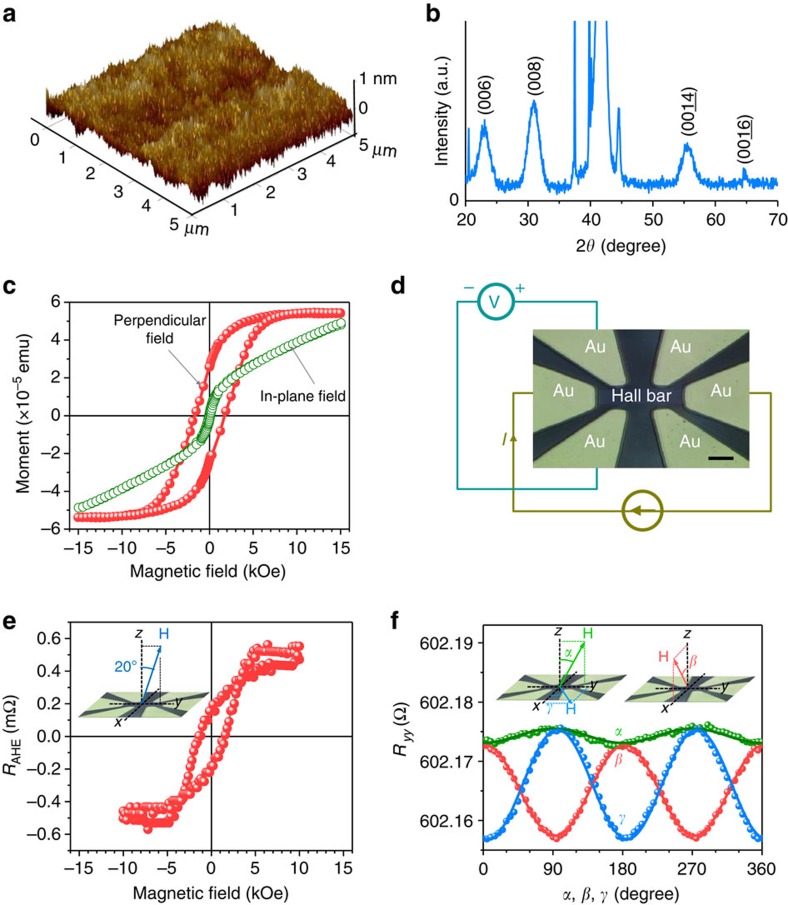
Properties of Pt/BaM films and Hall bar samples. (**a**) AFM surface image of the BaM film. The r.m.s. roughness is 0.17±0.02 nm. The roughness value is an average over the measurements on nine different 1 × 1 μm areas, and the uncertainty is the corresponding standard deviation. (**b**) XRD spectrum of the BaM film. (**c**) Magnetic hysteresis loops of the BaM film. (**d**) Optical image of the Pt(5 nm)/BaM(3 nm) Hall bar structure. Scale bar, 20 μm. (**e**) Anomalous Hall resistance *R*_AHE_ of the Hall bar measured as a function of a magnetic field. The inset is a schematic showing the magnetic field (**H**) direction which is in the *yz* plane and 20° away from the +*z* axis. (**f**) Angle-dependent longitudinal resistance *R*_*yy*_ of the Hall bar. The insets show the **H** directions for the *α*, *β* and *γ* scans. *α* is the angle of the field relative to +*z* in the *yz* plane, *β* is the angle of the field relative to the +*z* in the *xz* plane and *γ* is the angle of the field relative to +*x* in the *xy* plane. All the measurements were done at room temperature.

The data presented below were obtained using a Pt(5.0 nm)/BaM(3.0 nm) sample with the properties shown in Fig. 1. Figure 1a–c shows the properties of the BaM film, which was grown on a *c*-axis sapphire substrate by pulsed laser deposition^32^. The atomic force microscopy (AFM) image in Fig. 1a shows a uniform and smooth surface, and the analysis of the AFM data yielded a r.m.s. surface roughness of 0.17±0.02 nm. These results, together with other AFM data not shown, indicate that the BaM film has a reasonably good surface, which is critical for the realization of a high-quality Pt/BaM interface and a large SOT at the interface. Note that the roughness value here is an average over the measurements on nine different 1 × 1 μm areas, and the uncertainty is the corresponding s.d. The X-ray diffraction (XRD) spectrum in Fig. 1b indicates the *c*-axis orientation of the crystalline structure in the BaM film. The hysteresis loops in Fig. 1c were measured by a vibrating sample magnetometer with different field orientations, as indicated. The loops clearly show that the BaM film has perpendicular anisotropy, which confirms the *c*-axis orientation of the film. Analysis of the hysteresis data yielded an effective perpendicular anisotropy field *H*_a_=17.6 kOe, which is very close to the bulk value (17 kOe), and a saturation induction 4*πM*_s_=4.47 kG, which is slightly smaller than the bulk value (4.70 kG)^30^,^31^. These results together indicate that the BaM film is of high quality, although it is just slightly thicker than one unit cell of BaM materials (*c*=2.32 nm)^31^. Note that the loop shown in Fig. 1c for the perpendicular field is not as square as the loops of metallic films with perpendicular anisotropy^7^,^8^, and future work is of great interest that optimizes the growth conditions for the realization of BaM films with better crystalline orientations and higher squareness.

Figure 1d presents a photo image of a Pt(5.0 nm)/BaM(3.0 nm) Hall bar. The Pt layer was deposited on the BaM film by sputtering at room temperature, and the Pt/BaM Hall bar structure was fabricated by photolithography and argon ion milling. The central area of the Hall bar structure is 41 μm long and 11 μm wide, and the contact leads are made of a 400-nm-thick Au film with a 3-nm-thick Ti adhesion layer. With the electrical measurement configuration shown in Fig. 1d and a field configuration shown in Fig. 1e, one can measure the Hall resistance *R* of the Pt layer as a function of the field *H*. The obtained *R*(*H*) response consists of a linear background due to the ordinary Hall effect in the Pt layer and a hysteresis component due to the anomalous Hall effect (AHE) also in the Pt layer. Figure 1e presents the AHE resistance *R*_AHE_ as a function of *H*, which was obtained by subtracting the linear contribution from the initial *R*(*H*) data. One can see that the *R*_AHE_(*H*) data show a hysteresis loop response similar to the loop shown in Fig. 1c for a perpendicular field. This similarity indicates that *R*_AHE_ scales with the normal component of the magnetization (*M*_⊥_) in the BaM film, and one can probe the magnetization status in the BaM film by simply measuring *R*_AHE_(*H*).

Three additional points should be made about the data in Fig. 1e. First, the AHE response usually occurs in FMs only, and the presence of the AHE in the paramagnetic Pt film might be interpreted by the magnetic proximity effect (MPE)^33^,^34^ or the effects of the imaginary part of the spin mixing conductance at the Pt/BaM interface^35^,^36^, as in Pt thin films grown on Y_3_Fe_5_O_12_^37^,^38^. Figure 1f presents the angle dependence of the longitudinal resistance *R*_*yy*_ measured along the Hall bar length for the field rotating in three different planes. The *α* dependence clearly suggests the presence of the MPE in the Pt/BaM structure^33^,^34^, while the *β* dependence provides an evidence for the existence of the spin mixing conductance-associated magnetoresistance in the structure^35^,^36^,^37^. Note that the data in Fig. 1f also indicate that the *β* dependence is more noticeable than the *α* dependence, which might suggest that in the Pt/BaM sample the spin mixing conductance-associated magnetoresistance is larger than the MPE-produced magnetoresistance. Second, the planar Hall effect^39^,^40^ also appears in the Pt/BaM structure, as shown by the experimental data in Supplementary Note 1 and Supplementary Fig. 1. However, the planar Hall effect does not invalidate the use of AHE measurements to determine the magnetization status in the BaM. This is because all of the AHE measurements in this work involved the rotation of the magnetization in the *yz* plane, while the planar Hall effect concerns magnetization rotation in the *xy* plane. Note that there has been previous work that used the planar Hall effect to probe the magnetization in Y_3_Fe_5_O_12_ films, where, however, the magnetization rotated in the film plane due the absence of perpendicular anisotropy and the application of an in-plane field^41^. Finally, the remnant magnetization *M*_r_ and coercivity *H*_c_ indicated by the loop shown in Fig. 1e are both slightly smaller than the corresponding values indicated by the data in Fig. 1c. This is because the field had a small angle with the normal direction (20°) for the data in Fig. 1e but was along the normal direction for the data in Fig. 1c. One can also see that the loop in Fig. 1e appears noisier than that in Fig. 1c, which is because the AHE is usually very strong in FMs and is relatively weak in the paramagnetic Pt.

### Switching responses for out-of-plane magnetic fields

**Figure 2:**
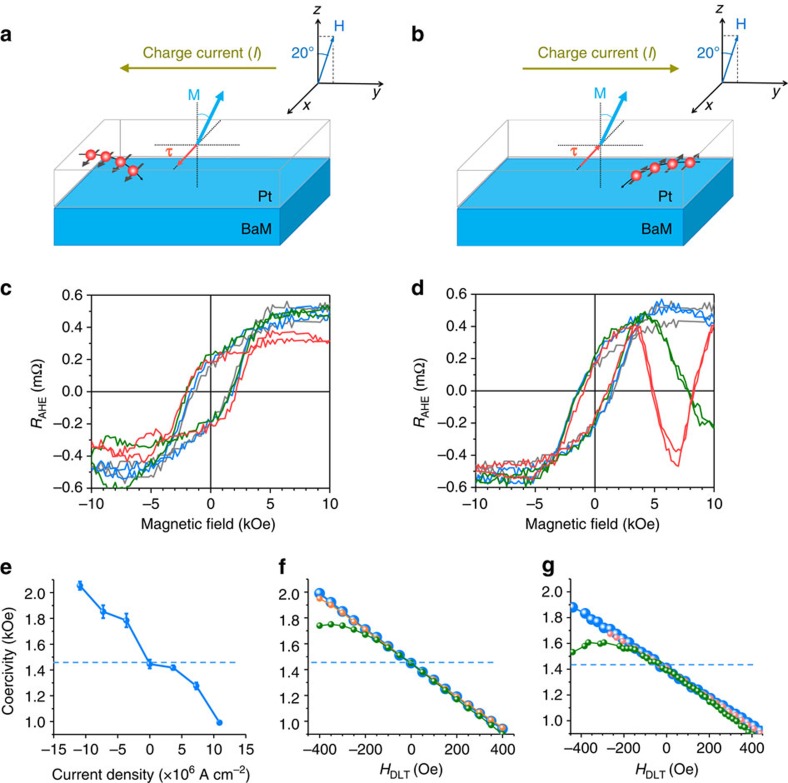
Switching responses in Pt/BaM for out-of-plane magnetic fields. (**a**,**b**) Effects of charge currents (*I*) in the Pt film on switching of the magnetization (**M**) in the BaM film under an out-of-plane field (**H**). The red spheres with arrows represent spin-polarized electrons deflecting toward the BaM layer. **M** represents the magnetization of BaM. **τ** represents the spin torque due to SHE. The direction of **H** is indicated in the insert. Note that the spin torque indicated in (**a**,**b**) is a damping-like torque (DLT). (**c**,**d**) Anomalous Hall resistance *R*_AHE_ of the Hall bar measured as a function of a magnetic field for different charge currents. The field was applied 20° away from the *z* axis, as shown in the insets of (**a**,**b**). In (**c**) Grey: *I*=0; Blue: *I*=−2 mA; Olive: *I*=−4 mA; and Red: *I*=−6 mA. In (**d**) Grey: *I*=0; Blue: *I*=2 mA; Olive: *I*=4 mA; and Red: *I*=6 mA. (**e**) Measured coercivity of the BaM film as a function of the charge current density. (**f**,**g**) Coercivity versus DLT field (*H*_DLT_) estimated for three different field-like torque (FLT) fields (*H*_FLT_) through macrospin and full micromagnetic simulations, respectively. Large blue spheres: *H*_FLT_=0; small red spheres: *H*_FLT_=*H*_DLT_/2; and small olive spheres: *H*_FLT_=*H*_DLT_. The dash line in (**e**–**g**) is the *H*_c_ at *I*=0. All the measurements were done at room temperature.

The magnetization switching indicated by the *R*_AHE_(*H*) data shown in Fig. 1e was driven mainly by the external field **H**; the currents used for the resistance measurements were low a.c. currents (0.8 mA) and their impact on the switching was negligible due to the a.c. nature of the current. However, if a large d.c. charge current is passed through the Pt layer, it can dramatically affect the switching in the BaM film. Figure 2 shows representative data that demonstrate this effect. Figure 2c,d shows the *R*_AHE_(*H*) responses measured for charge currents with opposite signs, as indicated. The *R*_AHE_(*H*) data were measured in the exact same way as described above for the data shown in Fig. 1e, but a large charge current was passed through the Hall bar right before each data point was taken. When the electrons flow in the Pt layer along the *y* axis, they produce a pure spin current flowing along the *z* axis due to the SHE^17^,^18^,^19^,^20^. For the current configuration shown in Fig. 2a, the SOT **τ** at the interface is along the +*x* direction, as indicated. This torque counters the torque produced by **H** and thereby hinders the switching of the magnetization **M** in the BaM film. This effect is clearly evident by the broadening of the *R*_AHE_(*H*) hysteresis loop with an increase in the current strength (*I*) as shown in Fig. 2c. In contrast, for the configuration shown in Fig. 2b, the SOT shares the same direction as the **H**-produced torque and thereby promotes the switching of **M** in the BaM film. This is shown by the narrowing of the *R*_AHE_(*H*) loop with an increase in the current strength in Fig. 2d.

Figure 2e plots the coercivity *H*_c_ as a function of the charge current density *J*_c_. Note that each point in the *R*_AHE_(*H*) loops shown in Fig. 2c,d shows an average over 10 measurements, and the error bars in Fig. 2e show the corresponding uncertainties for the averaging. The data in Fig. 2e indicate an *H*_c_ reduction of about 460 Oe for *J*_c_=1.09 × 10^7^ A cm^−2^ and an increase of about 610 Oe for *J*_c_=−1.09 × 10^7^ A cm^−2^.

The data also show an overall linear response for the current range used. This linear behaviour indicates that one can expect even bigger changes in *H*_c_ if larger currents are applied or thinner BaM films are used. When using large currents, however, attention should be paid to the Hall bar geometry and the electrical measurement configuration so that the bar would not be damaged by the Joule heating of the currents. Figure 2f,g presents the calculated coercivities which are discussed shortly. Note that for all of the data shown in Fig. 2, the field was tilted for about 20°, the same as for the data shown in Fig. 1e. The purpose of this was to break the symmetry of the magnetization in response to the field, enabling the demonstration of the SOT effects. Note also that the anomalous behaviour of the red and olive curves at high fields shown in Fig. 2d resulted from the drifting of the resistance measurement system.

### Switching responses for in-plane magnetic fields

**Figure 3:**
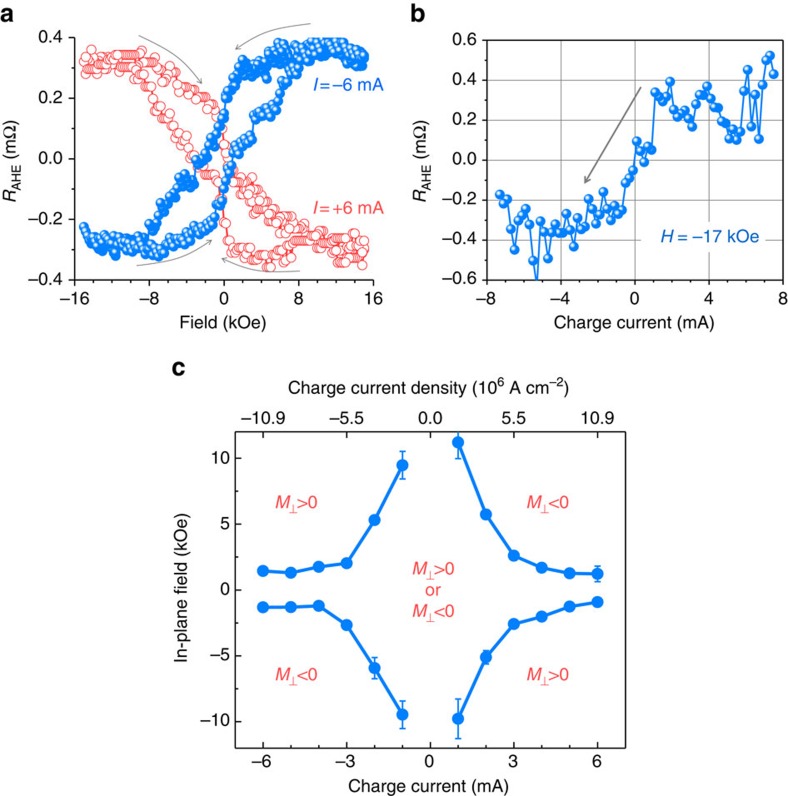
Switching responses in Pt/BaM for in-plane magnetic fields. (**a**) Anomalous Hall resistance *R*_AHE_ measured as a function of a magnetic field along the *y* axis for *I*=+6 mA and *I*=−6 mA, respectively. (**b**) *R*_AHE_ measured as a function of a charge current for a fixed field (*H*=−17 kOe) along the *y* axis. (**c**) Switching phase diagram where the data were obtained through the switching measurements similar to those shown in (**a**). In (**c**), *M*_⊥_>0 and *M*_⊥_<0 correspond to the normal component of the magnetization in the BaM film pointing up and down, respectively. The curves define the boundaries between different magnetization states. All the measurements were done at room temperature.

Figure 3 presents additional *R*_AHE_ data that further confirm the SOT effect demonstrated above. In contrast to the data in Fig. 2, all the data in Fig. 3 were taken with **H** in the film plane and along the Hall bar length. Figure 3a gives *R*_AHE_(*H*) loop responses measured under two charge currents with opposite signs. As indicated by the grey arrows, the two loops evolve in completely opposite manners. This result is consistent with previous switching experiments in other bi-layered systems^7^,^11^,^42^, and provides a strong evidence for the presence of the SOT at the interface. The physics underlying this result is as follows. When the strength of the in-plane field **H** is decreased gradually, the magnetic moments in the BaM film, which are initially in the film plane and along **H**, tend to tilt out of the film plane due to the strong perpendicular anisotropy. In the absence of charge currents, the moments do not favour tilting up or tilting down. In other words, the response of the moments to the field is symmetric about the film plane, and the normal component of the net magnetization in the film is zero, namely, *M*_⊥_=0. However, when a charge current is applied to the Pt film, the SOT (see **τ** in Fig. 2a,b) breaks the symmetry of the magnetization orientation in response to the field, resulting in *M*_⊥_≠0. The symmetry is broken in a different manner for the charge currents of opposite signs, and this gives rise to the opposite evolutions shown in Fig. 3a. Besides, the data in Fig. 3a also indicate that, when the in-plane field is reduced to zero, the up and down states of the net magnetization can be controlled by the charge current.

Figure 3b shows a *R*_AHE_ versus *I* response for a constant field applied along the Hall bar length direction (the −*y* direction). The data clearly indicate that one can use the charge current to switch between the *M*_⊥_>0 and *M*_⊥_<0 states with the assistance of an in-plane field. One can also see that *R*_AHE_ almost equals to zero at *I*=0, indicating the absence of hysteresis responses. This is consistent with the fact that in the absence of charge currents, **M** almost completely aligns with the in-plane field and *M*_⊥_ is almost zero.

Figure 3c gives the switching phase diagram for in-plane fields and charge currents. The data were obtained through the switching measurements similar to those shown in Fig. 3a. The diagram tells the charge current (or the magnetic field) required to switch **M** in the BaM film when a constant field (or a constant current) is applied. Note that each point in Fig. 3a shows the average over 10 measurements, and the error bars in Fig. 3c show the corresponding uncertainties for the averaging. The data in Fig. 3c seem to indicate that the uncertainties are relatively large for weak charge currents. This observation can be explained in terms of SOT-caused symmetry breaking discussed above. In brief, in the presence of a small charge current, when *H* is reduced to 0, one has *M*_⊥_ close to 0 due to small SOT. The net effect is that the *R*_AHE_(*H*) response has a small slope around *H*_c_ and the uncertainty in determining *H*_c_ is larger. In contrast, in the presence of a large charge current, the SOT is strong and *M*_⊥_ has a relatively large amplitude at *H*=0, which results in a larger slope in the *R*_AHE_(*H*) response around *H*_c_ and a relatively smaller uncertainty.

There are two additional points which should be discussed about the data shown in Fig. 3a. First, one would expect *M*_⊥_≈0 and *R*_AHE_≈0 at *H*=15 kOe since the magnetization should be almost saturated in the presence of an in-plane field of 15 kOe, as shown by the open circles in Fig. 1c. However, the data in Fig. 3a show non-zero *R*_AHE_ values at high fields. This is because the data were measured after the application of strong currents to the Pt layer, which produced a large SOT and thereby gave rise to either *M*_⊥_>0 or *M*_⊥_<0 even at high fields. Second, the two loops shown in Fig. 3a were obtained with in-plane fields, but they appear very different from the loop shown by the open circles in Fig. 1c that was also measured with in-plane fields. This is because the loop in Fig. 1c presents the in-plane component of **M** (*M*_||_), while the loops in Fig. 3a present the normal component of **M** (*M*_⊥_). One has *M*_||_=0 at *H*=0 for the *M*(*H*) loop measurements, and has *M*_⊥_≠0 at *H*=0 due to the SOT for the *R*_AHE_(*H*) loop measurements. These results are consistent with those observed for switching in topological insulator-based bi-layered systems^42^. They further confirm the presence of large SOTs at the Pt/BaM interface.

### Determination of SOT fields through macrospin and micromagnetic simulations

It is believed that the SOT in the Pt/BaM structure arises from the SHE^17^,^18^,^19^,^20^ in the Pt layer, as illustrated by the diagrams in Fig. 2a,b and as discussed previously for HM/FM systems^7^,^8^,^9^,^10^,^11^,^12^,^13^,^14^,^15^,^16^. In principle, the SHE can give rise to two different torques, a damping-like torque (DLT), which is indicated by the red arrows in Fig. 2a,b, and a field-like torque (FLT), which is not shown in the figures. Taking *H*_DLT_ and *H*_FLT_ as the corresponding effective fields of these two torques and following the analysis in Supplementary Note 2 and Supplementary Figs 2 and 3, one can write down the total field **H**_total_ on the normalized magnetization **m** in the BaM film as


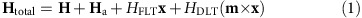


where **H** is the external field as indicated in Figs 1 and 2 and **x** is the unit vector along the +*x* direction. Note that both *H*_DLT_ and *H*_FLT_ are proportional to the charge current density *J*_c_ in the Pt.

It is also possible that the SOT in the Pt/BaM structure contains a contribution associated with the MPE^33^,^34^. As discussed above, the data shown in Fig. 1f suggest the presence of the MPE in the Pt/BaM structure. If the MPE presents, the SHE and the Rashba effect in the ferromagnetic-like Pt atomic layers would also give rise to a DLT and an FLT. It is fortunate that the corresponding *H*_DLT_ and *H*_FLT_ fields have the exactly same symmetry as the two SOT fields in equation (1). Further, they are also proportional to *J*_c_, as discussed in Supplementary Note 2. As a result, one can extract the strength of the SOT fields from the experimental data without having to know the relative contributions of the different mechanisms.

To determine the SOT field strength, simulations were carried out that were based on the Gilbert equation and used a macrospin model to represent the magnetization in the BaM film and the fields defined in equation (1). **H**_a_ was perpendicular to the BaM film plane. **H** was in the *yz* plane and was tilted 20° away from the +*z* direction initially, the same as in the experiment. The SOT fields *H*_FLT_ and *H*_DLT_ were obtained by comparing the experimental *H*_c_ values with those obtained from the simulations. This process involves three main steps as follows. First, one takes *J*_c_=0 and calculates *H*_c_. For this calculation, *H*_FLT_ and *H*_DLT_ are both set to zero, and the anisotropy field strength *H*_a_ is set in such a way that **m** flips when **H** is pointing in a direction opposite to its initial direction and has a strength equal to the experimentally measured *H*_c_ (1.45 kOe). Second, one considers the case of *J*_c_≠0 but takes *H*_FLT_=0 and uses simulations to find *H*_c_ values for given *H*_DLT_ values. From the point of view of the symmetry, it is clear that a flip in the direction of the *H*_FLT_ field does not lead to a change in *H*_c_ because the *H*_FLT_ field is orthogonal to **H**_a_, **H**, and **m**. In contrast, a flip in the direction of the *H*_DLT_ field breaks the symmetry and therefore affects *H*_c_. For this reason, as the first stage *H*_FLT_ is set to zero and *H*_c_ is calculated as a function of *H*_DLT_ in this step. Third, one sets *H*_FLT_ to a non-zero value and repeats the simulations to find *H*_c_ values for given *H*_DLT_ and *H*_FLT_ combinations. Note that the adjustment of *H*_a_ to make the *H*_c_ value comparable to the experimental value for the *J*_c_=0 case is done for convenience of comparison of the measured and calculated results. The rate of the change of *H*_c_ with *H*_DLT_, however, remains a quantity which is independent of this approximation. Supplementary Note 3 discusses the details of the simulations.

In Fig. 2f, the blue dots show the *H*_c_ versus *H*_DLT_ response calculated for *H*_FLT_=0, and the other dots are discussed shortly. One can see that the *H*_c_ versus *H*_DLT_ response shows a linear dependence, the same as the experimental *H*_c_ versus *J*_c_ data. Specifically, *H*_c_ increases to about 2.0 kOe when *H*_DLT_ is −400 Oe and decreases to about 0.95 kOe when *H*_DLT_ is 400 Oe. The same change in the experimental *H*_c_ value is observed when *J*_c_ changes between −10^7^ A cm^−2^ and 10^7^ A cm^−2^. Thus, one can conclude that the strength of *H*_DLT_ in the Pt/BaM is about 400 Oe at *J*_c_=10^7^ A cm^−2^. For HM/FM systems, previous work observed a *H*_DLT_ field of 17 Oe for Pt(2 nm)/Co(0.6 nm)/AlO_*x*_ (ref. 7), 50 Oe for Pt(3 nm)/Co(0.6 nm)/Al_2_O_3_(2 nm)^43^, 55–200 Oe for Pt(3 nm)/Co(0.9 nm)/Ta(0.5–4 nm)^13^, 50 Oe for Pt(3 nm)/Co_80_Fe_20_(0.6 nm)/MgO (ref. 9) and 200 Oe for Ta(5 nm)/Co_80_Fe_20_(0.6 nm)/MgO (ref. 9), all corresponding to the same charge current density *J*_c_=10^7^ A cm^−2^. One can see that the *H*_DLT_ field in the Pt/BaM is stronger than those previously reported values.

The above-described analysis assumed *H*_FLT_=0, and similar analysis was carried out for *H*_FLT_≠0. The red and olive dots in Fig. 2f show the *H*_c_ versus *H*_DLT_ responses calculated for *H*_FLT_=*H*_DLT_/2 and *H*_FLT_=*H*_DLT_, respectively. It is evident from the data in Fig. 2f that the effect of *H*_FLT_ is almost negligible for *H*_FLT_=*H*_DLT_/2. For *H*_FLT_=*H*_DLT_, the *H*_c_ versus *H*_DLT_ response deviates from the linear dependence for strong negative charge currents, which, however, has not been observed experimentally, indicating that *H*_FLT_ is relatively small in the Pt/BaM structure.

The simulations discussed above used a macrospin to represent **m** in the BaM film. In the experiment, however, the switching of **m** may not be realized through coherent rotation, but through domain nucleation and subsequent domain wall motion, thanks to the relatively large size of the Pt/BaM Hall bar sample. In consideration of this possibility, full micromagnetic simulations were also carried out that used the well-established OOMMF code with the Oxs_SpinXferEvolve module^44^ to numerically solve Supplementary equation (9). The *H*_DLT_ field defined by Supplementary equation (8) or (13) was taken into account by an equivalent spin torque with the polarization along the *x* axis. The simulated film size was set to 1 × 1 μm and the mesh size was set to 5 × 5 × 3 nm. As in the experiments, **H** was in the *yz* plane and was tilted 20° away from the +*z* direction initially. The procedures for the determination of the SOT fields were the same as those described above for the macrospin simulations, and the results are presented in Fig. 2g. By comparing Fig. 2f,g, one can see that the results from the two simulations are close to each other for all three different *H*_FLT_ fields, confirming the accuracy of the macrospin simulation-yielded *H*_DLT_ fields described above. One can also see that, for a given *H*_DLT_, *H*_c_ from the micromagnetic simulation are slightly smaller (about 4%) than that from the macrospin model. This means that, for a given *H*_c_ change, the corresponding *H*_DLT_ field from the micromagnetic analysis is slightly larger than that from the macrospin analysis. Finally, it should be noted that the micromagnetic simulations indicated that the switching in the BaM was realized through domain nucleation and growth.

The numerical work presented above clearly indicates that the SOT in the Pt/BaM structure is larger than that in the HM/FM systems. This conclusion can also be obtained by direct comparisons of experimental data. For example, a reduction in *H*_c_ of about 440 Oe was observed for a Pt(3 nm)/Co(0.6 nm)/AlO_*x*_ structure for an increase in *J*_c_ of 10^7^ A cm^−2^ (ref. 45), while a very similar *H*_c_ reduction can be seen from the data in Fig. 2e. If one considers that the BaM layer (3 nm) in this work is five times thicker than the Co layer (0.6 nm) in the previous work, one can conclude that the SOT efficiency in the Pt/BaM structure is indeed higher than that in the Pt/Co structure. Further, in the Pt/Co structure there is always a portion of the applied current flowing in the Co layer and being wasted, which is a weakness in terms of energy efficiency.

It is important to highlight that this work may have far-reaching implications for the future development of spin-torque electronics. Specifically, the HM/MI systems offer a number of benefits in comparison with the HM/FM structures studied previously^7^,^8^,^9^,^10^,^11^,^12^,^13^,^14^,^15^,^16^. First, perpendicular anisotropy in MI films originates from bulk intrinsic anisotropy^30^,^31^ rather than surface anisotropy. This means that, when being used for actual devices, MI films have no constrains on the thickness, unlike the FM film counterpart that often relies on surface anisotropy to realize perpendicular anisotropy^46^. Second, the charge current flows in the HM layer only, not in the MI layer. In contrast, in a HM/FM structure the charge current also flows in the FM, resulting in certain parasitic effects. The advantage of no shunting current in the MI film becomes particularly important when the HM layer is replaced by a topological insulator layer. Recent work demonstrates that topological insulators may produce considerably larger SOT than HM materials^42^,^47^. Finally, the magnetic damping is usually significantly lower in MIs than in FMs. For example, the intrinsic Gilbert damping constant in BaM materials is 7 × 10^−4^ (refs 30, 31), which is at least 10 times smaller than the value in Permalloy. This advantage is significant for spin-torque oscillator applications, where the current threshold for self-oscillations decreases with the damping^48^ as well as for logic device applications that require low-damping, insulating spin channels^49^,^50^. As discussed above, the SOT strength in the Pt/BaM structure was found to be higher than the value reported previously for HM/FM systems^7^,^9^,^13^,^43^. This result is surprising considering the fact that there are almost no electron diffusions at the interface from the Pt layer to the BaM film, unlike in the HM/FM counterpart.

In summary, strong SOT effects have been observed in a Pt/BaM bi-layered structure. It is found that the charge current applied to the Pt film can switch the normal component of the magnetization in the BaM film between the up and down states when an in-plane magnetic field is applied. When an out-of-plane field is applied to switch the BaM film, the charge current can reduce or increase the switching field by as much as about 500 Oe. The results demonstrate the presence of large SOT in HM/MI systems in comparison to HM/FM systems and the possibility of efficient SOT-induced switching in HM/MI systems, thereby presenting potential direction for the future development of magnetic memory and logic devices for energy-efficient computing. Future work is of great interest that demonstrates current-driven, rather than current-assisted, switching in narrow BaM strips and nanoscale BaM elements. Toward this end, approaches that could be taken include replacing Pt with materials with substantially stronger spin–orbit coupling^8^,^42^,^47^, such as topological insulators; reducing the anisotropy in the BaM via doping, for example, scandium^51^; and utilizing thinner BaM films.

## Methods

### Material growth

The BaM film was grown on a *c*-axis sapphire substrate by pulsed laser deposition (PLD) techniques^32^. During the deposition, the oxygen pressure was 300 mTorr, the substrate temperature was 800 °C, the substrate-to-target separation was fixed at 4 cm and the energy fluence of the laser beam was set to 0.7 J cm^−2^. The laser pulse repetition rate was increased from 1 to 5 pulse(s) per second in five equal steps over the first 5 min and was then set to 10 pulses per second for the remaining deposition. After the deposition, the substrate was cooled down at a rate of −2 °C min^−1^ in 400 Torr oxygen. The sample was then annealed at 850 °C for 4 h in a standalone tube furnace, with a heating rate of 10 °C min^−1^ and a cooling rate of −2 °C min^−1^.

### Device fabrication

The 5.0-nm-thick Pt film was deposited on the BaM film in an AJA magnetron sputtering system. The Pt (5.0 nm)/BaM (3.0 nm) film stack was patterned into an 11-μm-wide Hall bar with a photolithography system first and then etched in an argon ion milling system. The Hall bar contact leads were made of Ti(3.0 nm)/Au(400 nm). They were fabricated by the photolithography, thermal evaporation and lift-off processes.

### Measurements

The roughness of the BaM film was measured with a Veeco Innova atomic force microscope. The crystalline structure and film thickness were measured with a Rigaku Smartlab XRD/XRR system. The hysteresis loops of the film were measured with a Microsense EV7 vibrating sample magnetometer. The electrical measurements were conducted with a Quantum Design physical property measurement system supplemented with two lock-in-amplifiers, a preamplifier and two Keithley metres.

### Data availability

The data that support the findings of this study are available from the corresponding author upon request.

## Additional information

**How to cite this article:** Li, P. *et al*. Spin–orbit torque-assisted switching in magnetic insulator thin films with perpendicular magnetic anisotropy. *Nat. Commun.* 7:12688 doi: 10.1038/ncomms12688 (2016).

## Supplementary Material

Supplementary Information Supplementary Figures 1-3, Supplementary Notes 1-3 and Supplementary References
