# Supplementary material for: Spin–orbit torque-assisted switching in magnetic insulator thin films with perpendicular magnetic anisotropy
Source: Nat Commun. 2016 Sep 1;7:12688. doi: 10.1038/ncomms12688 (PMC5025772; doi:10.1038/ncomms12688)
Supplement: Supplementary Information — Supplementary Figures 1-3, Supplementary Notes 1-3 and Supplementary References [file ncomms12688-s1.pdf]

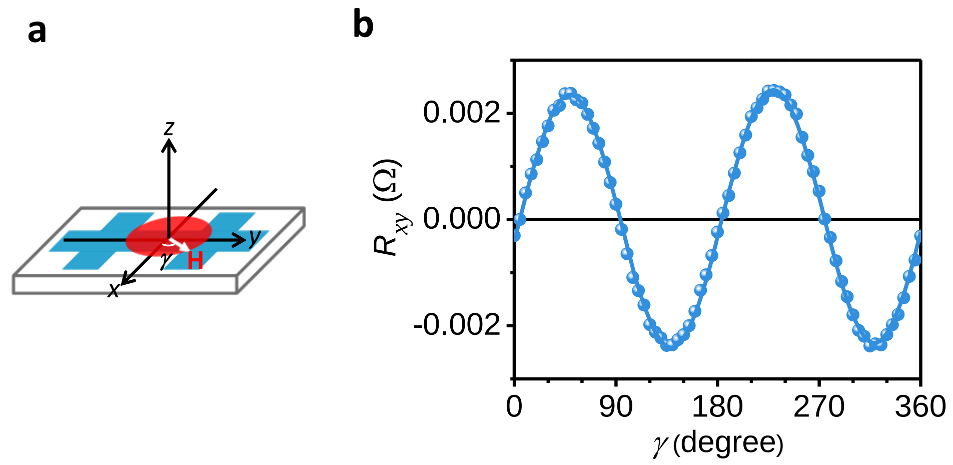

Supplementary Figure 1. Planar Hall effect resistance as a function of the angle of an in-plane field. **a**, Schematic of the planar Hall resistance measurement setup. The magnetic field (**H**) is applied in the film plane at angle γ with the *x* axis. **b**, Angle-dependent measurement of resistance R_{xy} vs. angle γ.

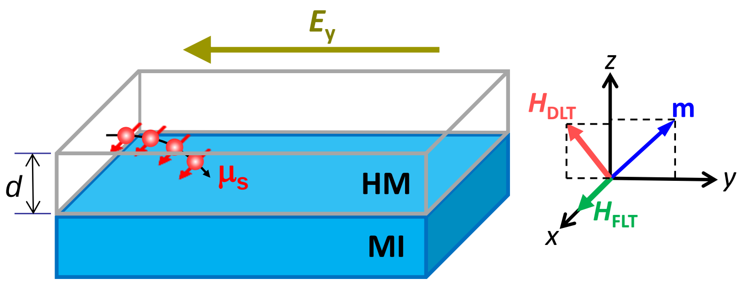

Supplementary Figure 2. Schematic illustration of SHE-associated SOT fields. HM denotes heavy metal and MI denotes magnetic insulator. $\boldsymbol{\mu}_s$ and E_y denote spin accumulation and the electrical field associated with the charge current, respectively. The red spheres with arrows represent spin-polarized electrons deflecting toward the MI layer. The inset shows the coordinate. **m** denotes the magnetization, and H_{DLT} and H_{FLT} denote the effective fields of the damping-like torque (DLT) and the field-like torque (FLT), respectively.

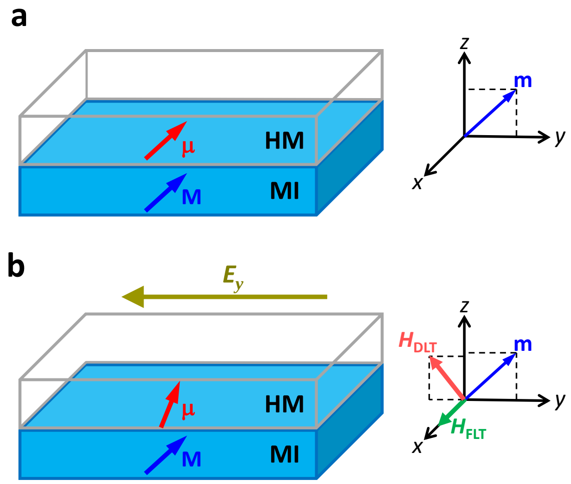

Supplementary Figure 3. Schematic illustration of MPE-associated SOT fields. a, in the absence of a charge current the MPE-induced magnetic moment **μ** is parallel to and thereby exerts no torque on the **m** of the MI. **b**, in the presence of a charge current **μ** exerts a torque on **m**. The insets show the coordinates. **m** denotes the magnetization, and H_{DLT} and H_{FLT} denote the effective fields of the damping-like torque (DLT) and the field-like torque (FLT), respectively.

Supplementary Note 1. Planar Hall Effect in Pt/BaM

In addition to the ordinary Hall effect and the anomalous Hall effect (AHE), the planar Hall effect (PHE)[1,2] is also present in the Pt/BaM structure. Supplementary Figure 1 presents the PHE resistance R_{xy} of the same Hall bar as described in the text measured with an in-plane field as a function of the angle of the field (γ) relative to the +*x* direction. One can see that the PHE resistance shown in Supplementary Figure 1 is about one order of magnitude larger than the AHE resistances presented in the text. However, the planar Hall effect does not invalidate the use of AHE measurements to determine the magnetization status in the BaM film. This is because all of the AHE measurements in this work involved the rotation of the magnetization in the *yz* plane, while the planar Hall effect concerns magnetization rotation in the *xy* plane.

Supplementary Note 2. Origins of Spin-Orbit Torque in Pt/BaM

The experimental data presented in the main text clearly demonstrate the existence of spin-orbit torque (SOT) at the Pt/BaM interface upon the application of a direct current in the Pt layer. It is very likely that this SOT arises from the spin Hall effect (SHE)-produced spin accumulation in the Pt near the Pt/BaM interface, as discussed previously for non-magnetic heavy metal/ferromagnetic metal heterostructures. It is also possible that there exist magnetic proximity effect (MPE)-induced magnetic moments in several Pt atomic layers near the Pt/BaM interface, as indicated by the experimental data shown in Fig. 1f in the main text. In those ferromagnetic-like Pt atomic layers, the SOT can appear through the SHE and the Rashba effect, just as in ferromagnetic metals. The sections below discuss in detail these two different SOT mechanisms. The discussions consider a more general system, a non-magnetic heavy metal (HM)/magnetic insulator (MI) bi-layered structure, but are applicable to the particular Pt/BaM structure concerned in this work.

a. SHE-Associated SOT

Supplementary Figure 2 depicts the essence of the SHE-associated SOT mechanism. When there is no charge current in the HM layer, SOT is zero and the normalized magnetization **m** in the MI layer remains in its equilibrium state. When a charge current is passing through the HM layer, the SHE produces a pure spin current that is polarized along the *x* axis and flows along the *z* axis, as shown schematically in the Supplementary Figure 2. The net effect is the presence of spin accumulation $\boldsymbol{\mu}_s$ near the HM/MI interface. This spin accumulation can exert torques on **m** through the *s-d* exchange interaction at the interface.[3]

Based on the Onsager theory,[4] one can correlate $\boldsymbol{\mu}_s$ to the spin current density **J** with the following spin diffusion equation

$$\mathbf{J}(z) = -\frac{\sigma}{2e}\frac{\partial \boldsymbol{\mu}_s(z)}{\partial z} + \sigma\theta_{\mathrm{SH}}E_y\mathbf{x} \qquad (1)$$

where *e* is the electron charge, σ is the electrical conductivity of the HM, θ_{SH} is the spin Hall angle in the HM, and E_y is the applied electric field. Assuming that λ is the spin diffusion length in the HM, Supplementary Equation (1) has the following solution

$$\boldsymbol{\mu}_s(z) = \mathbf{C}_1 e^{z/\lambda} + \mathbf{C}_2 e^{-z/\lambda} \qquad (2)$$

where the vector coefficients $\mathbf{C}_1$ and $\mathbf{C}_2$ can be obtained using the following two boundary conditions:

(i) The spin current density vanishes at the vacuum interface of the HM layer, namely, $\mathbf{J}(z=d)=0$.

(ii) The spin current density at the HM/MI interface is proportional to the spin mixing conductance $G_{\uparrow\downarrow}$ and can be written as $e\mathbf{J}(z=0) = \mathrm{Re}[G_{\uparrow\downarrow}]\mathbf{m}\times[\mathbf{m}\times\boldsymbol{\mu}_s(z=0)] + \mathrm{Im}[G_{\uparrow\downarrow}]\mathbf{m}\times\boldsymbol{\mu}_s(z=0)$.

The final expression for the spin accumulation at the HM/MI interface reads

$$\boldsymbol{\mu}_s(z=0) = \mu_s^{(0)}\mathbf{x} + a\mu_s^{(0)}\mathbf{m}\times(\mathbf{m}\times\mathbf{x}) + b\mu_s^{(0)}(\mathbf{m}\times\mathbf{x}) = \mu_s^{(0)}(1-a)\mathbf{x} + b\mu_s^{(0)}(\mathbf{m}\times\mathbf{x}) \tag{3}$$

where $\mu_s^{(0)}$ denotes the spin accumulation in the absence of the *s-d* exchange interaction and is given by

$$\mu_s^{(0)} = 2e\theta_{\mathbf{SH}}\lambda E_y \tanh\left(\frac{d}{2\lambda}\right) \tag{4}$$

and the coefficients *a* and *b* are given by

$$a = \mathrm{Re}\left[\frac{2\lambda G_{\uparrow\downarrow}\coth\left(\frac{d}{\lambda}\right)}{\sigma + 2\lambda G_{\uparrow\downarrow}\coth\left(\frac{d}{\lambda}\right)}\right] \tag{5}$$

$$b = \mathrm{Im}\left[\frac{2\lambda G_{\uparrow\downarrow}\coth\left(\frac{d}{\lambda}\right)}{\sigma + 2\lambda G_{\uparrow\downarrow}\coth\left(\frac{d}{\lambda}\right)}\right] \tag{6}$$

Through the interfacial *s-d* exchange interaction, the spin accumulation $\boldsymbol{\mu}_s(z=0)$ exerts an effective field on **m**, which is given by

$$\mathbf{H}_{\mathrm{SOT}} \propto J_{sd}\mu_s^{(0)}(1-a)\mathbf{x} + J_{sd}b\mu_s^{(0)}(\mathbf{m}\times\mathbf{x}) \tag{7}$$

where J_{sd} is the *s-d* exchange constant. The field along $\mathbf{x}$ gives rise to a field-like torque (FLT), while the one along $\mathbf{m}\times\mathbf{x}$ leads to a damping-like torque (DLT) or the so-called Slonczewski torque.[5] These two fields are referred as H_{FLT} and H_{DLT}, respectively, in the discussions below.

The total field $\mathbf{H}_{\mathrm{total}}$ on **m** in the MI layer can be then written as

$$\mathbf{H}_{\mathrm{total}} = \mathbf{H} + \mathbf{H}_a + H_{\mathrm{FLT}}\mathbf{x} + H_{\mathrm{DLT}}(\mathbf{m}\times\mathbf{x}) \tag{8}$$

where **H** and $\mathbf{H}_a$ are the external magnetic field and the effective anisotropy field in the MI, respectively. With this total field, one can then calculate the magnetization dynamics in the MI layer by numerically integrating the Gilbert equation

$$\frac{\partial\mathbf{m}}{\partial t} = -\frac{|\gamma|}{1+\alpha^2}\left[\mathbf{m}\times\mathbf{H}_{\mathrm{total}} + \alpha\mathbf{m}\times(\mathbf{m}\times\mathbf{H}_{\mathrm{total}})\right] \tag{9}$$

where $|\gamma|$ is the absolute gyromagnetic ratio and α is the Gilbert damping constant.

b. MPE-Associated SOT

The MPE-associated SOT mechanism is illustrated schematically in Supplementary Figure 3. When there is no charge current in the HM layer, the MPE-induced magnetic moment $\boldsymbol{\mu}$ in the HM must be collinear with the normalized magnetization **m** in the MI layer, as shown in Supplementary Figure 3a. As a result, there is no torque on **m** in the MI. Once a charge current is applied to the HM layer, the SHE and the Rashba effect give rise to a SOT field that exerts on $\boldsymbol{\mu}$ in the Pt and thereby tilts $\boldsymbol{\mu}$ away from its initial equilibrium direction. The net effect is that $\boldsymbol{\mu}$ in the HM is not collinear with **m** in the MI anymore and $\boldsymbol{\mu}$ exerts a torque on **m**, as shown schematically in Supplementary Figure 3b.

The SOT field in the ferromagnetic-like HM produced by the SHE and the Rashba effect can be written as[6,7]

$$\mathbf{H}_{\mathrm{SOT}} = h_{\mathrm{FLT}}\mathbf{x} + h_{\mathrm{DLT}}\left(\mathbf{m}\times\mathbf{x}\right) \tag{10}$$

where h_{FLT} and h_{DLT} are the effective fields corresponding to the field-like torque and the damping-like torque, respectively. Considering that the conduction electrons in the HM respond to external stimuli much faster than the magnetization **m** in the MI, one can then write **μ** in the HM as

$$\boldsymbol{\mu} = \chi\left[\mathbf{H} + J_{sd}\mathbf{m} + h_{\mathrm{FLT}}\mathbf{x} + h_{\mathrm{DLT}}\left(\mathbf{m}\times\mathbf{x}\right)\right] \tag{11}$$

where χ is the magnetic susceptibility of the HM and $J_{\mathrm{sd}}\mathbf{m}$ denotes the interfacial *s-d* exchange field. One can see from Supplementary Equation (11) that **μ** is not collinear with **m** due to the SOT fields. Through the *s-d* exchange interaction, **μ** in the HM will also exert an effective field on **m** in the MI, which is $J_{\mathrm{sd}}\boldsymbol{\mu}$. Thus, the total field $\mathbf{H}_{\mathrm{total}}$ on **m** can be written as

$$\mathbf{H}_{\mathrm{total}} = \mathbf{H} + \mathbf{H}_a + J_{sd}\boldsymbol{\mu} \approx \mathbf{H} + \mathbf{H}_a + J_{sd}\chi h_{\mathrm{FLT}}\mathbf{x} + J_{sd}\chi h_{\mathrm{DLT}}\left(\mathbf{m}\times\mathbf{x}\right) \tag{12}$$

where the term $J_{\mathrm{sd}}\chi\mathbf{H}$ is dropped out because it is much smaller than **H**, and the term $(J_{\mathrm{sd}})^2\chi\mathbf{m}$ is also dropped out because it is along **m** and thus does not contribute the dynamics of **m**. Assuming $H_{\mathrm{FLT}} = J_{sd}\chi h_{\mathrm{FLT}}$ and $H_{\mathrm{DLT}} = J_{sd}\chi h_{\mathrm{DLT}}$, Supplementary Equation (12) can be rewritten as

$$\mathbf{H}_{\mathrm{total}} \approx \mathbf{H} + \mathbf{H}_a + H_{\mathrm{FLT}}\mathbf{x} + H_{\mathrm{DLT}}\left(\mathbf{m}\times\mathbf{x}\right) \tag{13}$$

By comparing Supplementary Equations (8) and (13), one can conclude that the SOT fields in the two different mechanisms actually have the exactly same symmetry. As a result, one can extract the strength of the SOT fields from the experimental data without having to know the relative contributions of the different mechanisms. The details on the estimation of the SOT fields are provided in the next section. It should be emphasized that $\mu_s^{(0)}$ in Supplementary Equation (4) and h_{FLT} and h_{DLT} in Supplementary Equation (10) are all proportional to the charge current density J_{c}. This means that the strength of the SOT fields is also proportional to J_{c}.

Supplementary Note 3. Estimation of SOT Fields in Pt/BaM

In this section the strength of the SOT fields in the Pt/BaM structure is estimated and is compared to that in HM/ferromagnetic metal (FM) bi-layered systems. In a HM/FM system, both H_{FLT} and H_{DLT} are proportional to the charge current density J_{c}, no matter whether they originate from the SHE or the Rashba effect.[6,7] This is the same in the Pt/BaM structure (see Supplementary Note 2). This fact enables the comparison between the SOT efficiency in the Pt/BaM and that in the HM/FM systems studied in previous work.

a. Macrospin Simulations

To determine the SOT field strength, simulations are carried out that use a macrospin **m** to represent the magnetization in the BaM film and numerically solve Supplementary Equation (9). The simulations use the fields defined in Supplementary Equations (8) or (13). The anisotropy field $\mathbf{H}_a$ is perpendicular to the BaM film plane. The external field **H** is in the *yz* plane and is tilted 20 degrees away from the +*z* direction initially, the same as in the experiment (see Fig. 1e). The SOT fields H_{FLT} and H_{DLT} are unknown and will be obtained by comparing the experimental coercivity values with the values obtained from the simulations.

One first starts from calculating the coercivity H_{c} for the charge current density J_{c}=0. For this calculation, one takes H_{FLT}=0 and H_{DLT}=0; the strength of $\mathbf{H}_{\mathrm{a}}$ is set in such a way that **m** in the BaM flips when **H** is pointing in a direction opposite to its initial direction and has a strength equal to the experimentally measured H_{c} value, which is 1.45 kOe. Then one considers the case of $J_{\mathrm{c}}\neq 0$ and finds H_{c}

values for given H_{FLT} and H_{DLT} values via numerical simulations. Upon the application of a charge current in the Pt layer, both H_{FLT} and H_{DLT} present and contribute to the dynamics of **m**. However, from the point of view of the symmetry it is clear that a flip in the direction of the H_{FLT} field does not lead to a change in H_c because the H_{FLT} field is orthogonal to $\mathbf{H}_a$, **H**, and **m**. In contrast, a flip in the direction of the H_{DLT} field breaks the symmetry and therefore affects H_c. For this reason, as the first step H_{FLT} is set to zero and H_c is calculated as a function of H_{DLT}. It is important to note that the adjustment of the anisotropy field to make the H_c value comparable to the experimental value for the J_c=0 case is done for convenience of comparison of the measured and calculated results. The rate of the change of H_c with H_{DLT}, however, remains a quantity which is independent of this approximation.

Figures 2e and 2f in the manuscript compare the calculated results with the experimental data. Figure 2e shows the experimental H_c vs. J_c data. In Figure 2f, the blue dots show the H_c vs. H_{DLT} response calculated for H_{FLT}=0, and the other dots are discussed shortly. One can see that the H_c vs. H_{DLT} response shows a linear dependence, the same as the experimental H_c vs. J_c data. Specifically, H_c increases to about 2.0 kOe when H_{DLT} is -400 Oe and decreases to about 0.95 kOe when H_{DLT} is 400 Oe. The same change in the experimental H_c value is observed when J_c changes between -10^7 A·cm^{-2} and 10^7 A·cm^{-2}. Thus one can conclude that the strength of H_{DLT} in the Pt/BaM is about 400 Oe at $J_c=10^7$ A·cm^{-2}. For HM/FM systems, previous work observed a H_{DLT} field of 17 Oe for Pt(2 nm)/Co(0.6 nm)/AlO_x,[8] 50 Oe for Pt(3 nm)/Co(0.6 nm)/Al_2O_3(2 nm),[9] 55-200 Oe for Pt(3 nm)/Co(0.9 nm)/Ta(0.5-4 nm),[10] 50 Oe for Pt(3 nm)/$Co_{80}Fe_{20}$(0.6 nm)/MgO,[11] and 200 Oe for Ta(5 nm)/ $Co_{80}Fe_{20}$(0.6 nm)/MgO, [11] all corresponding to the same charge current density $J_c=10^7$ A·cm^{-2}. One can see that the H_{DLT} field in the Pt/BaM is stronger than those previously reported values. Considering that (1) the BaM layer (3 nm) in this work is at least three times thicker than the FM layer (<1 nm) in the previous work and (2) in an HM/FM structure there is always a portion of the applied current flowing in the FM layer and being wasted, one can conclude that the SOT efficiency in the Pt/BaM structure is indeed higher than that in the HM/FM systems.

The above-described analysis assumed H_{FLT}=0, and similar analysis can be carried out for $H_{FLT}\neq0$. The red and olive dots in Figure 2f show the H_c vs. H_{DLT} responses calculated for $H_{FLT}= H_{DLT}/2$ and $H_{FLT}= H_{DLT}$, respectively. It is evident from the data in Figure 2f that the effect of H_{FLT} is almost negligible for $H_{FLT}= H_{DLT}/2$. For $H_{FLT}= H_{DLT}$, the H_c vs. H_{DLT} response deviates from the linear dependence for strong negative charge currents, which, however, has not been observed experimentally, indicating that H_{FLT} is relatively small in the Pt/BaM structure.

b. Micromagnetic Simulations

The simulations described above used a macrospin to represent the magnetization **m** in the BaM film. In the experiment, however, the magnetization switching in the BaM film may not be realized through coherent rotation, but through domain nucleation and subsequent domain wall motion, thanks to the relatively large size of the Pt/BaM Hall bar sample. In consideration of this possibility, full micromagnetic simulations were also carried out to estimate the SOT fields in the Pt/BaM structures.

Specifically, the simulations used the well-established OOMMF code to numerically solve Supplementary Equation (9). The code is based on the Oxs_SpinXferEvolve module, which is intended for the simulation of Slonczewski-type torques.[12] The H_{DLT} field, as defined by Supplementary Equations (8) or (13), was taken into account by an equivalent spin torque with the polarization along the *x* axis, enabling the modeling of the spin-orbit torque as if it was a spin-transfer torque. In order to break the symmetry and accelerate the simulations, the magneto-crystalline anisotropy field value and the anisotropy easy axis direction were randomized by a few percent and a few degrees, respectively. The simulated film size was set to 1 μm × 1 μm, and the mesh size was set to 5 nm x 5nm x 3 nm. As in the experiments, the external field was in the *yz* plane and was tilted 20° away from the +*z* direction initially. The procedures

for the determination of the SOT fields were the same as those described above for the macrospin simulations.

Figure 2g in the main text presents the results obtained from the micromagnetic simulations in the same format as in Figure 2f. By comparing Figures 2f and 2g, one can see that the results from the two simulations are close to each other for all three different H_{FLT} fields, confirming the accuracy of the macrospin simulation-yielded H_{DLT} fields described above. One can also see that, for a given H_{DLT}, the coercivity values from the micromagnetic simulation are slightly smaller (about 4%) than those from the macrospin model. This means that, for a given coercivity change, the corresponding H_{DLT} field from the micromagnetic analysis is slightly larger than that from the macrospin analysis, suggesting that the micromagnetic simulation indicates the presence of slightly stronger SOT in Pt/BaM than the macrospin simulation.

It should be noted that H_{c} can depend on many extrinsic factors, such as film surface roughness, defects, and anisotropy distribution. However, for a given film sample, these factors are fixed and will not change upon the application of a current in the Pt layer, thus it is valid to examine the SOT strength by checking the effects of the strength and polarity of the charge current in the Pt film on the H_{c} value of the BaM film.

Supplementary References

1. Ky, V. D. Planar Hall effect in ferromagnetic films. *Phys. Stat. Sol*. **26**, 565 (1968).

2. Naftalis, N. *et al*. Field-dependent anisotropic magnetoresistance and planar Hall effect in epitaxial magnetite thin films. *Phys. Rev. B* **84**, 094441 (2011).

3. Takahashi, S., Saitoh, E. & Maekawa, S. Spin current through a normal-metal/insulating ferromagnet junction. *J. Phys.: Conf. Ser*. **200**, 062030 (2010).

4. Chen, Y.-T. *et al*. Theory of spin Hall magnetoresistance. *Phys. Rev. B* **87**, 144411 (2013).

5. Slonczewski, J. C. Current-driven excitation of magnetic multilayers. *J. Magn. Magn. Mater*. **159**, L1–L7 (1996).

6. Khvalkovskiy, A. V. *et al*. Matching domain-wall configuration and spin-orbit torques for efficient domain-wall motion. *Phys. Rev. B* **87**, 020402(R) (2013).

7. Lee, O. J. *et al*. Central role of domain wall depinning for perpendicular magnetization switching driven by spin torque from the spin Hall effect. *Phys. Rev. B* **89**, 024418 (2014).

8. Liu, L., Lee, O. J., Gudmundsen, T. J., Ralph, D. C. & Buhrman, R. A. Current-induced switching of perpendicularly magnetized magnetic layers using spin torque from the spin Hall effect. *Phys. Rev. Lett*. **109**, 096602 (2012).

9. Garello, K. et al. Symmetry and magnitude of spin–orbit torques in ferromagnetic heterostructures. *Nat. Nanotech*. **8**, 587-593 (2013).

10. Woo, S., Mann, M., Tan, A. J., Caretta, L. & Beach, G. S. D. Enhanced spin-orbit torques in Pt/Co/Ta heterostructures. *Appl. Phys. Lett*. **105**, 212404 (2014).

11. Emori, S., Bauer, U., Ahn, S., Martinez, E. & Beach, G. S. D. Current-driven dynamics of chiral ferromagnetic domain walls. *Nat. Mater*. **12**, 611-616 (2013).

12. OOMMF User's Guide, Version 1.0, M. J. Donahue and D. G. Porter, Interagency Report NISTIR 6376, National Institute of Standards and Technology, Gaithersburg, MD (Sept 1999).
